# iPSC-derived retinal organoids: Biological insights and progress toward photoreceptor replacement therapy

**DOI:** 10.1016/j.xcrm.2026.103053

**Published:** 2026-09-25

**Authors:** Akiko Maeda, Xiaoxu Han, Michiko Mandai

**Affiliations:** 1 Research Center, Kobe City Eye Hospital, Hyogo 650-0047, Japan; 2 Department of Ophthalmology, Peking Union Medical College Hospital, Chinese Academy of Medical Sciences and Peking Union Medical College, Beijing 100730, China; 3 RIKEN Center for Biosystems Dynamics Research, Hyogo 650-0047, Japan; 4 Research Organization of Science and Technology, Ritsumeikan University, Shiga 525-8577, Japan

**Keywords:** retinal organoid, transplantation, retinal degeneration, genome editing, regenerative therapy, photoreceptor replcement, multiple electrode array, clinical study

## Abstract

Induced pluripotent stem cell (iPSC)-derived retinal organoids (ROs) have emerged as a platform for photoreceptor replacement therapy, supported by two complementary approaches: cone precursor cell transplantation and RO transplantation. Both have demonstrated proof of concept for functional synaptic integration with the host retinal network, with the first clinical application of iPSC-derived RO transplantation conducted in 2020. Key mechanistic insights include the role of the host environment in cone integration, genome editing-based enhancement of host-graft synaptogenesis recapitulating developmental synaptic assembly, and synaptic rewiring capacity in the adult primate retina. Beyond biology, successful clinical translation requires establishing meaningful endpoints that reflect stepwise therapeutic improvement, scalable good manufacturing practice (GMP)-compliant manufacturing including validated preservation protocols, and regulatory frameworks accommodating progenitor-stage cell therapies. Together, these advances offer a realistic path toward vision restoration in patients with currently untreatable retinal degenerative diseases.

## Graphical abstract

**Figure:**
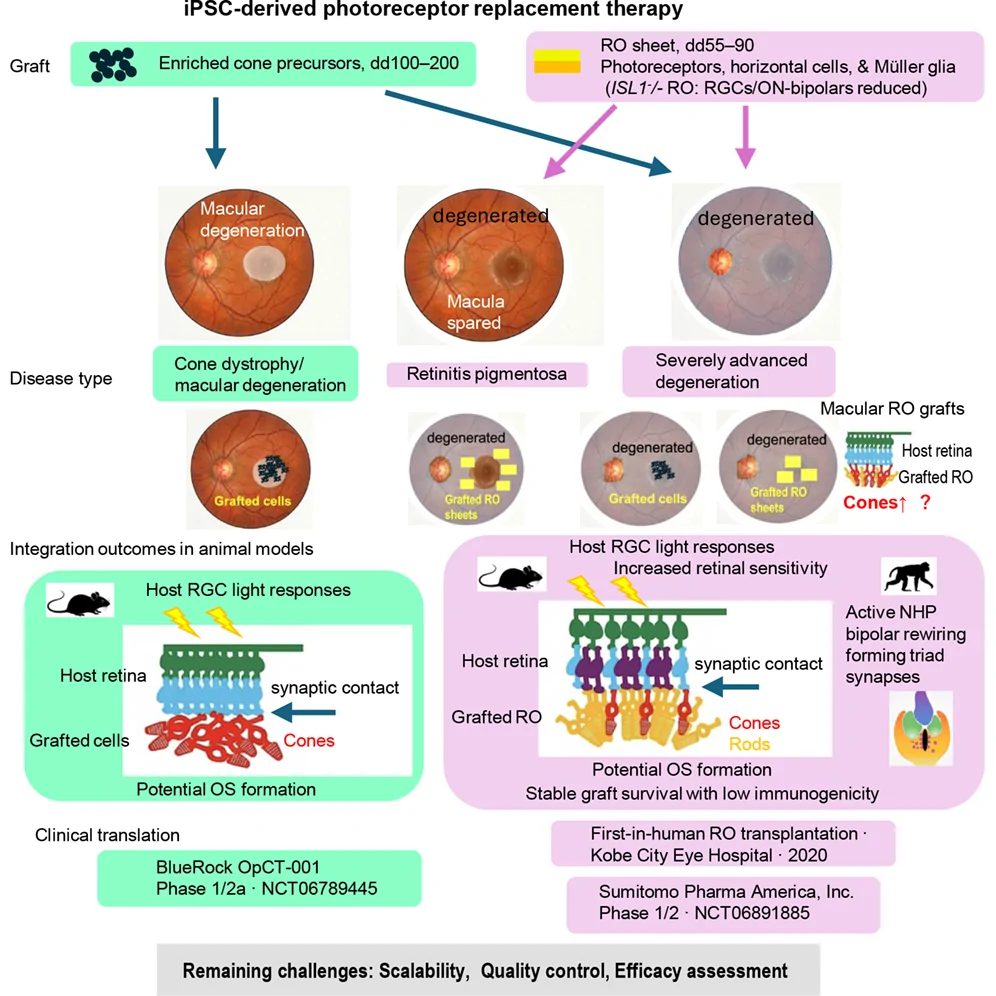

## Teaser

Mandai et al. review iPSC-derived photoreceptor replacement therapy, contrasting cone precursor and retinal organoid transplantations. They highlight host-dependent cone integration, gene-edited organoids for enhanced integration, and synaptic rewiring in the adult primate retina, providing a framework to restore vision in patients with untreatable retinal degenerations.

## Introduction

Inherited retinal diseases (IRDs) and other retinal degenerative disorders represent a major cause of irreversible blindness worldwide. The retina, as a part of the central nervous system, was classically considered to have no regenerative capacity once degenerated. Despite substantial advances in gene discovery, the translation of genetic insights into effective therapies has remained challenging, in part, due to the limited availability of human retinal tissues and the incomplete fidelity of animal models in recapitulating human-specific disease processes.

The advent of induced pluripotent stem cell (iPSC) technology has provided a transformative approach to overcome these limitations.^1^ Combined with retinal organoid (RO) technology,^2^^,^^3^ patient-specific iPSCs can now be differentiated into ROs and retinal pigment epithelium (RPE), retinal ganglion cells (RGCs), retinal assembloids,^4^ and other three-dimensional ocular models, enabling the reconstruction of key aspects of human retinal architecture and function *in vitro*. These advances have greatly expanded opportunities for studying retinal biology and developing regenerative therapies. In parallel, iPSC-derived retinal tissues have also enabled major progress in disease modeling, mechanistic studies, and therapeutic screening.^5^^,^^6^^,^^7^^,^^8^^,^^9^^,^^10^^,^^11^^,^^12^ Among the various applications of RO technology, cell replacement therapy has emerged as one of the most clinically advanced and potentially transformative strategies for restoring vision in patients with retinal degeneration.

In this review, we focus specifically on the development of photoreceptor replacement therapy using iPSC-derived ROs. We summarize the key biological and technological advances that have driven the field, from early studies investigating donor cell integration and host-graft interactions to recent progress in retinal sheet transplantation and the first clinical applications of iPSC-derived ROs. While ROs have also become powerful platforms for disease modeling, drug discovery, and gene therapy evaluation, these rapidly expanding areas are beyond the scope of the present review. Instead, we highlight the translational pathway toward photoreceptor restoration and discuss the remaining challenges and future prospects for achieving functional vision recovery through retinal cell transplantation.

## Retinal structures in view of functional recovery

Diseases of the anterior segment of the eye have become largely treatable owing to their accessibility and the long-standing development of cataract surgery and corneal transplantation. This leaves retinal neuronal diseases, including retinal and macular degeneration, diabetic retinopathy, and glaucoma—the latter characterized by progressive RGC degeneration—as the major causes of blindness in developed countries. The retina is structurally organized into three cell nuclear layers: a photoreceptor layer (outer nuclear layer [ONL]) that first responds to light; an inner nuclear layer (INL) consisting of bipolar cells as secondary neurons, amacrine cells, horizontal cells, and Müller glia; and the inner-most RGC layer that transmits visual information to the brain through the RGCs’ axons, which form the optic nerve. Two synaptic layers are interposed between these nuclear layers: the outer plexiform layer (OPL), situated between the ONL and INL, and the inner plexiform layer (IPL), between the INL and the RGC layer. The RPE forms a monolayer of cells opposed to the photoreceptors, providing essential support for their function (Figure 1A). RPE supports the clearance and renewal of photoreceptor outer segments (OSs) through phagocytosis, recycling of visual pigments, and secretion of neurotrophic factors such as pigment epithelium-derived factor (PEDF) for photoreceptors and vasular endothelial growth factor (VEGF) for choroidal capillary maintenance.^13^

**Figure 1:**
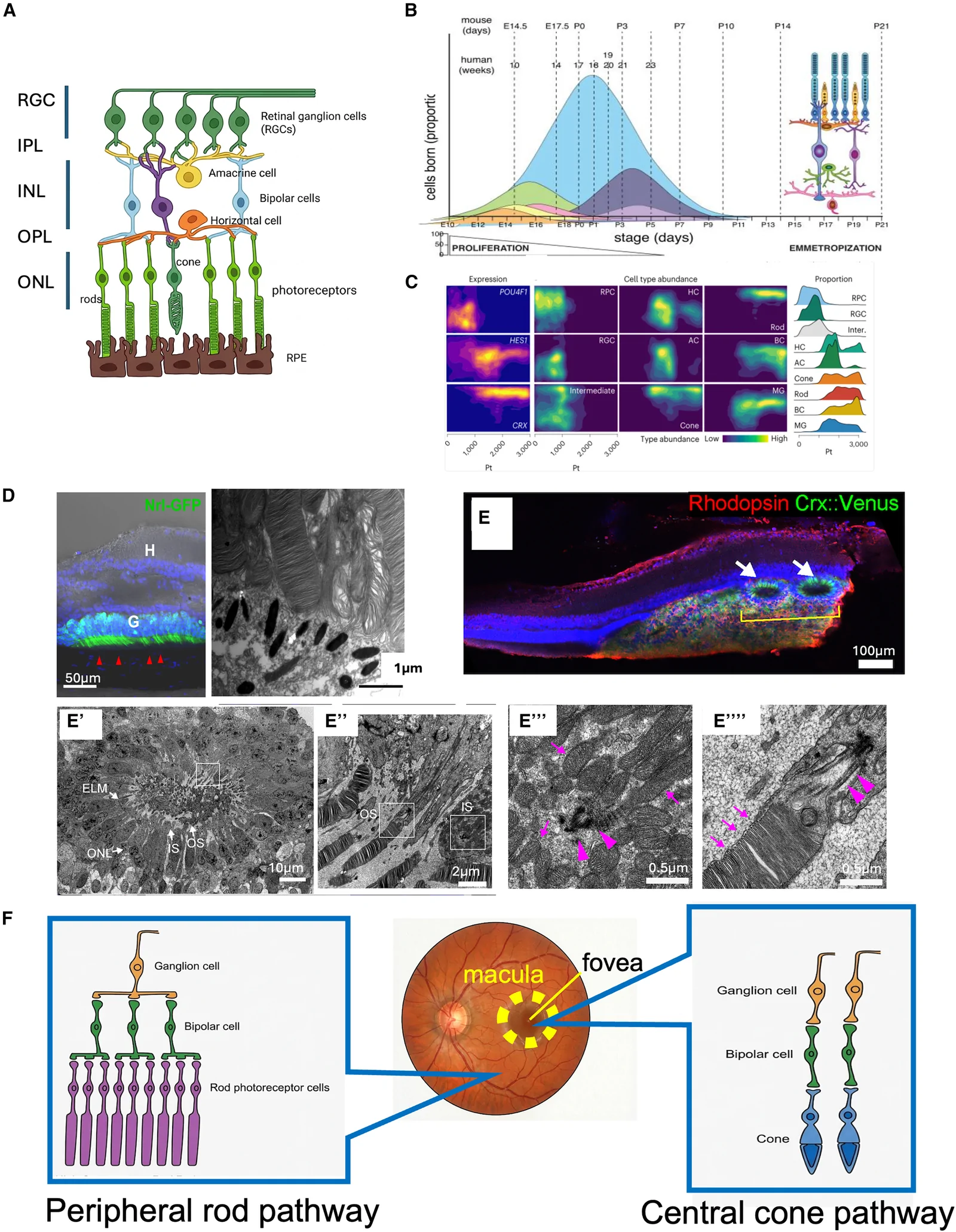
Retinal cell organization, developmental timeline, and recapitulation in retinal organoids (A) Major retinal cell types involved in visual signal transmission are illustrated. Photoreceptors comprise an outer nuclear layer (ONL) and transmit signals to the inner nuclear layer (INL), which consists of second-order neurons including bipolar cells and signal-modulating interneurons (amacrine and horizontal cells), and ultimately to retinal ganglion cells (RGCs), which relay the integrated visual output to the brain via long axons that collectively form the optic nerve. (B) Schematic for the sequential birth order of retinal cell types during development. RGCs and horizontal cells are generated first, followed by amacrine cells, cone photoreceptors, and bipolar cells, with rod photoreceptors and Müller glia born last. Corresponding differentiation timing in mouse (Mu) and human (Hu) retina is indicated by dashed lines. Adapted from Aldiri et al.^14^ (C) Retinal organoid differentiation recapitulates the *in vivo* developmental sequence. Shown is a representative spatial and temporal gene expression analysis of RGC (*POU4F1*), Müller glia (*HES1*), and photoreceptor (*CRX*) markers across the inner-to-outer structural axis (left: spatial distribution of each cell type) and pseudotime temporal axis (right: proportional abundance of each cell type over pseudotime), demonstrating that cell type emergence in ROs follows the order observed during human retinal development (adapted from Wahle et al.^15^). (D) Correlative fluorescence and electron microscopy of mouse iPSC-derived RO grafts transplanted into *rd1* retina. Right: outer segments (OSs) formed by Nrl-GFP-positive grafted photoreceptors in the same section as shown in the left, where OSs are indicated by red arrowheads. H, host; G, graft. Adapted from Assawachananont et al.^16^ (E–E′′′') Outer segment development in human ESC-derived RO grafts transplanted into retinal degeneration nude rats. (E) Fluorescence image showing CrxVenus (green)-positive photoreceptor rosettes (arrows). (E′) Low-magnification electron micrograph of the same section showing ONL rosette structure with inner segments (ISs) and OSs below the external limiting membrane (ELM). (E′′) Higher-magnification views from boxed area in (E′) showing organized OS disc membranes. (E′′′ and E′′′′) Further magnified views from boxed areas in (E″) showing the IS containing mitochondria (arrows), connecting cilia (arrowheads) (E′′′), and stacked disks (arrows) (E′′′′). Adapted from Shirai et al.^17^ (F) The macula is the central region of the fundus, dominated by cone photoreceptors, with the highest spatial resolution achieved at the central fovea. This high acuity is supported by a near 1:1:1 ratio of cones-to-bipolar cells-to-RGCs. In contrast, the peripheral retina is predominantly composed of rod photoreceptors, which converge onto bipolar cells and RGCs at high ratios, enabling sensitive vision under dark or dim-light conditions.

In the field of retinal regenerative therapy, photoreceptors, RPE cells, and RGCs have been the major targets. In this review, we specifically focus on photoreceptor replacement therapies, which have made considerable progress over the past decade, with key mechanistic insights and promising preclinical outcomes based on two major approaches— cone precursor cell transplantation and RO transplantation. Cone precursor cell transplantation aims to use enriched photoreceptor populations delineating other retinal cell types, while ROs contain not only photoreceptor cells but also support retinal cell types, including Müller glia and horizontal cells, collectively referred to as “an intrinsic cellular scaffold,” which facilitates photoreceptor maturation and functional integration utilizing the developing RO environment. Building on this body of preclinical evidence, the first clinical study using iPSC-derived RO sheets, conducted in 2020, demonstrated stable graft survival, potential photoreceptor development by *in vivo* imaging, and mild transient improvements in some visual tests in a patient with visual acuity of hand motion.^18^ In IRDs characterized by primary photoreceptor degeneration, the remaining retinal cells including secondary neurons, bipolar cells, tertiary neurons, and RGCs are preserved for a period following photoreceptor loss, providing a therapeutic window during which replacement therapy may enable partial functional reconstruction. Among alternative therapeutic approaches such as retinal prosthesis and optogenetics, cell-based therapy offers a potential advantage that lies in its capacity to engage the existing host retinal network. Bipolar cells, the secondary neurons to receive input from photoreceptors, have a number of subtypes that transmit “on” and “off” light responses, coordinate with other retinal cells such as horizontal cells and amacrine cells together, and elaborately tune visual information such as light intensity, background light adaptation, contrast, and temporal resolutions for transmission to the brain (Figure 1A).^19^^,^^20^ In replacement therapies for photoreceptor loss, how to incorporate grafted photoreceptors into the remaining host inner retinal network would be the key to the degree of functional restoration.

Although many IRDs involve pathogenic variants in photoreceptor-specific genes—causing primary photoreceptor loss—some IRDs manifest photoreceptor degeneration secondary to functional failure of RPE cells, as in diseases caused by alterations in *LRAT*,^21^ *RPE65*,^22^ and *MERTK*.^23^ This makes it necessary for some diseases—the ones with RPE-related causal genes and those with advanced pathology where both RPE and photoreceptors cells degenerate—to supply not only the photoreceptors but also RPE cells in combination to properly restore visual function.

## Background for photoreceptor cell replacement therapy

Cell-based therapies for photoreceptor loss can trace back to fetal retinal transplantation. Preclinical studies have demonstrated potential synaptic integration and signal transmission from transplanted retinas, as recorded in the superior colliculus.^24^^,^^25^^,^^26^ Safety and stable survival of grafted fetal retinas or retinal cells were observed in some clinical studies, with a low risk of immune rejection, and potential post-transplant visual effect, mostly transient, was also reported in some patients.^27^^,^^28^^,^^29^ A major concern was the presence of inner retinal layers in fetal retinal grafts, which may impede synaptic integration between host inner retinal neurons and grafted photoreceptors. A detailed study in a mouse model of congenital stationary night blindness (*Gnat1*^−/−)^ further demonstrated substantial functional integration of early postnatal photoreceptors into the host retina.^30^ These studies gained momentum with two milestone technologies: iPSC reprogramming^1^ and RO technology.^2^^,^^3^ Notably, however, a paradigm-shifting discovery was the finding of material transfer, whereby several groups simultaneously reported that what was once interpreted as functional photoreceptor integration was, in fact, a transfer of cytoplasmic materials from the grafted photoreceptors to the remaining host photoreceptors.^31^^,^^32^^,^^33^ Since then, the field has adopted more cautious interpretations and careful experimental approaches. These include (1) the confirmation of genuine donor cell integration using sex-mismatched transplantation combined with X or Y chromosome *in situ* hybridization to distinguish donor from host cell nuclei,^34^^,^^35^^,^^36^^,^^37^ (2) the use of end-stage retinal degeneration models, where the near-absence of remaining host photoreceptors ensures that evident functional recovery is most likely attributable to the graft,^34^^,^^35^^,^^36^^,^^37^^,^^38^ and (3) in the case of RO transplantation, retention of photoreceptors within the organized structure of the graft, such that synaptic connections formed with host cells can be unambiguously attributed to graft-derived photoreceptors.^38^ Despite these complexities, the field has continued to advance, and evidence for genuine photoreceptor integration and functional restoration has accumulated across multiple experimental models. The following sections describe the current state of cell-based approaches to photoreceptor replacement in further detail.

## ROs are strong tools for robust photoreceptor differentiation

Photoreceptor differentiation was initially achieved by recapitulating developmental processes and inducing the eye field in culture^39^^,^^40^^,^^41^; however, RO technology enabled more robust and reproducible retinal cell production. The fundamental principle underlying RO generation is the self-organization of sequentially specified ocular cell populations directed from pluripotent stem cells.^2^^,^^3^^,^^42^ These RO structures accompany the formation of ciliary body-like structure, the progenitor source of retinal cells.^43^ Generally, RO development is quite consistent, with each retinal cell type differentiating in a sequential order that recapitulates *in vivo* retinal development, in which inner retinal cells and cone photoreceptor cells are generated first followed by rod photoreceptors and bipolar cells, with full maturation occurring later.^14^ Photoreceptor maturation generally requires more than 200 days in culture,^15^^,^^44^ and advanced maturation *in vitro* remains challenging to achieve. ROs can develop photoreceptors bearing rudimentary OSs that exhibit light responsiveness *in vitro*.^44^ On the other hand, more advanced OS morphological maturation, including elongated OS with organized disc membranes, has been observed following transplantation into animal eyes, as demonstrated by electron microscopy (Figures 1D and 1E)^16^^,^^17^ and supported by the restoration of light responses in transplanted retinas from several sources.^34^^,^^35^^,^^36^^,^^38^^,^^45^ It has been consistently observed that ocular *in vivo* environment strongly facilitates photoreceptor maturation, as demonstrated by accelerated transcriptomic maturation of transplanted photoreceptors compared with age-matched cultured controls by single-cell RNA sequencing (scRNA-seq),^46^ quantitative increases in synaptic markers following transplantation,^47^ and support of laminar structuring even from non-layered retinal spheroids.^48^ Nevertheless, key environmental factors responsible for such maturation have not been determined, and their identification would strongly advance the application of RO technology *in vitro*.

## Cone or rod photoreceptor replacement in animal models in context of diseases

Cones dominate the central part of the human retina and contribute to color vision and visual resolution, as determined by visual acuity. In contrast, rods contribute to retinal sensitivity, allowing for vision under dim light, and occupy a larger area of the retina outside macula (Figure 1F). High visual acuity in the fovea relies on the near one-to-one connectivity between cones, bipolar cells, and RGCs, whereas high retinal sensitivity under dim-light conditions is achieved through the high convergence of rods onto rod bipolar cells and subsequently RGCs.^20^ Retinitis pigmentosa (RP) is the major group of IRDs with primarily rod degeneration, and the initial symptoms represent night blindness and peripheral vision loss. Cones are considered to undergo secondary degeneration following rod loss^49^ through several proposed mechanisms: a bystander effect mediated by gap junctions between dying rods,^50^ microglial toxicity,^51^ depletion of rod-derived trophic factors such as rod-derived cone viability factor (RdCVF),^52^ and oxidative stress resulting from excess oxygen availability following the loss of the large rod photoreceptor population.^53^ For these conditions, rod reconstruction prior to cone loss may restore retinal sensitivity, some peripheral vision, and could potentially slow secondary cone degeneration. On the other hand, some IRDs that primarily involve cone degeneration including Stargardt disease and cone dystrophy, and more environmental diseases such as age-related macular degeneration (AMD), cause central vision impairment, with visual acuity loss from early stages. For this disease type, restoration of macular cone pathway would be the major therapeutic approach.

In the preclinical studies worldwide, the trend is now categorized into two approaches. One is cell suspension transplantation, initially aiming for rod reconstruction in stationary night blindness model (*Gnat1*^−/−^) or RP mice such as *rd1* (*Pde6b*) that primarily lose rods followed by cones,^54^^,^^55^ but more recently, especially after the discovery of cytoplasmic transfer between rod photoreceptors, directly focusing on cone pathway reconstruction using enriched cone precursor cells for models such as end-stage rd1, cone dystrophy (Cpfl1), and LCA-4 (Aipl1).^34^^,^^35^^,^^36^ Enriched cone precursor cell suspension is well suited for low-convergent photoreceptor-to-bipolar cell reconstruction and is also adaptable to ongoing photoreceptor degeneration, such as conditions in which host rod photoreceptors remain but cones are primarily lost. Engrafted cone precursors have been shown to form synaptic contacts with host inner retinal neurons in mice, as detailed in a separate section. The other approach is RO transplantation, which initially aims for rod-based reconstruction outside the macula (Figure 2A),^17^^,^^56^ whereas a recent study showed cone-dominant engraftment in the primate macula,^57^ suggesting that the graft photoreceptor composition may be tailored to the transplantation site. In typical RP, there is considerable time, probably spanning decades, until the remaining cone function is completely lost following rod degeneration.^58^ In the presence of supportive neighboring cells such as horizontal cells and Müller cells within the graft, RO grafts stably integrate with the retained photoreceptor layer structure, with aligned synaptic ribbons ready to form synaptic contacts with host bipolar cells. Details are described in separate sections.

**Figure 2:**
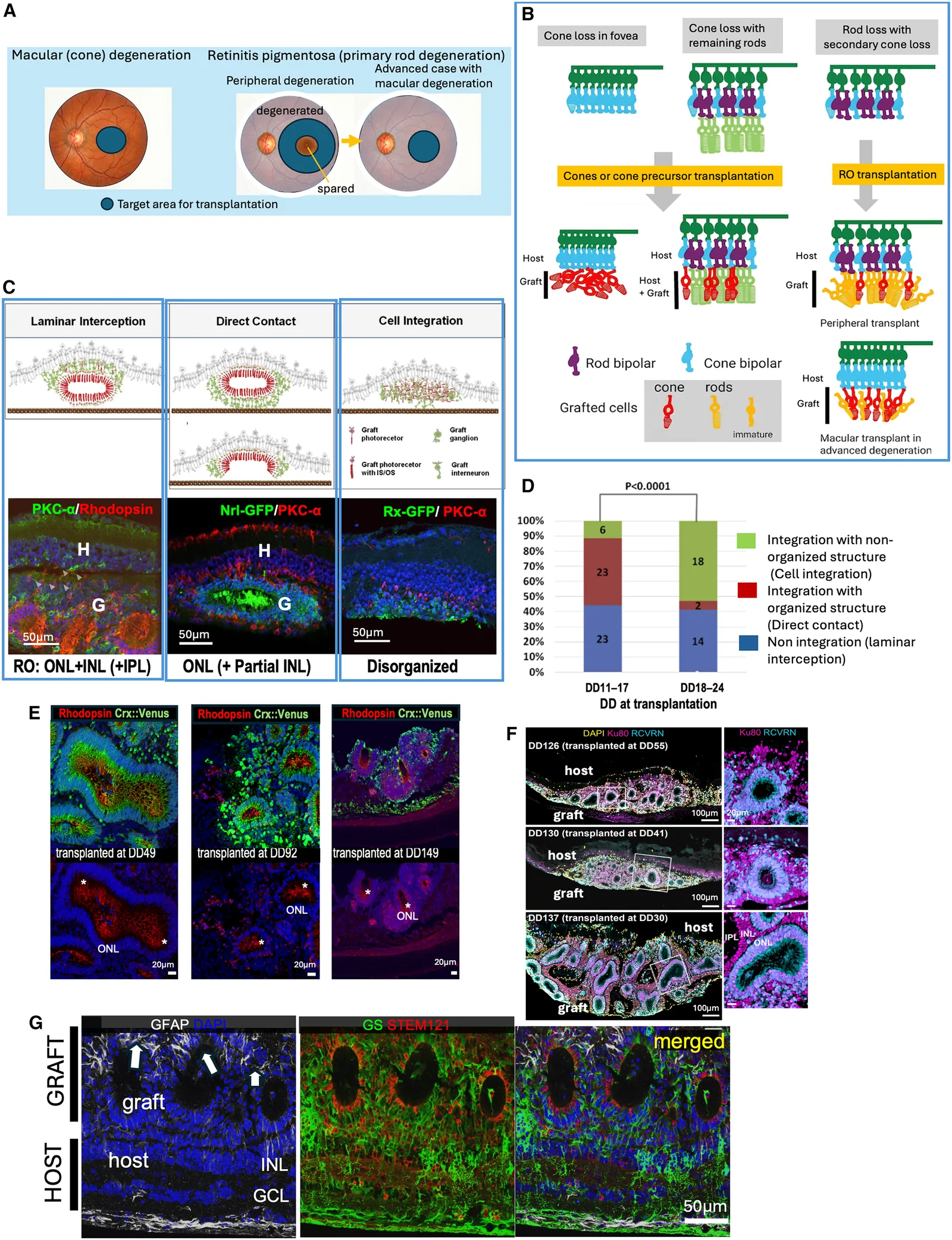
Disease-based rationale and cellular targets for cone precursor and retinal organoid transplantation, and developmental stage-dependent structural integration of retinal organoid grafts (A) Target areas for transplantation in macular cone degeneration and peripheral rod degeneration (retinitis pigmentosa), including advanced cases with secondary macular involvement. (B) Top: three representative disease conditions are illustrated: cone loss in the fovea, cone loss in the presence of surviving host rods, and rod-dominant degeneration with secondary cone loss as seen in typical retinitis pigmentosa. Bottom: schematic illustrations of hypothetical graft-host integration outcomes in the human disease context, inferred from preclinical experiments in mouse models (cone transplantation) and non-human primate models (RO transplantation). (Left two frames) Cone precursor transplantation into end-stage cone-degenerated macula yields unstructured but mature cone integration (mouse model,^36^), whereas transplantation of relatively mature cones in the presence of surviving host rods supports more structured donor cone integration and maturation (mouse model^35^). (Right two frames) In extramacular RO transplantation, graft cones and rods integrate in a rod-dominant manner to form synaptic contact with host bipolar cells (NHP model^17^^,^^56^), whereas macular RO transplantation in advanced degeneration yields a higher proportion of engrafted cone photoreceptors, possibly reflecting cone-favoring properties of the macular microenvironment (NHP model^57^). Color coding: rod bipolar cells, purple; cone bipolar cells, blue; grafted cones, red; grafted rods, yellow. (C) Schematic illustrations of three integration patterns observed following RO transplantation into end-stage *rd1* retinas: laminar interception, in which the inner retinal structure of the graft interposes between the graft ONL and host INL, limiting photoreceptor integration; direct contact, in which the graft ONL directly abuts the host INL with potential for synaptic interaction; and cell integration, in which individual graft cells contact the host INL without retaining ONL structure. Graft ONL often forms rosettes. Bottom: representative immunohistological images of each pattern. Scale bars: 20 μm. Adapted from Assawachananont et al.^16^ (D) Nearly half of the graft ONL rosettes show the direct contact pattern when transplanted at dd11-17 (adapted from Assawachananont et al.^16^). *p* value calculated by chi-square test. (E) Human ES cell-derived ROs (CrxVenus reporter) transplanted at different DDs into retinal degeneration nude rats. Earlier-stage ROs developed thicker INL and ONL following transplantation. CrxVenus labels grafted photoreceptors and subsets of bipolar cells, with rhodopsin-positive outer segment-like structures within the luminal space of the rosettes. Adapted from Shirai H et al.^17^ (F) Monkey iPSC-derived RO grafts transplanted at different DDs into retinal degeneration nude rats, shown at comparable post-transplant time points (magnified views of boxed areas are shown on right). Grafts transplanted at DD30 (bottom) developed thick IPL/INL adjacent to ONL rosettes. Adapted from Uyama H et al.^59^ (see supplemental information). (G) Immunofluorescence images of the graft-host interface showing GFAP (white), glutamine synthetase (GS, red), and the human cell marker STEM121 (green) with DAPI counterstain. Despite the presence of GS-positive Müller cells throughout the graft, GFAP upregulation is absent at integrating sites but prominent in the non-integrating regions (arrows). Scale bars: 50 μm. Adapted from Tu et al.^56^ ONL, outer nuclear layer; INL, inner nuclear layer; IPL, inner plexiform layer.

### Preclinical evidence for cone photoreceptor replacement in mouse IRD models

Post-mitotic rod-mediated photoreceptor transplantation had been reported as a promising strategy for visual function recovery,^30^ until the discovery of cytoplasmic material transfer between the donor and host rod photoreceptors following cell suspension transplantation,^31^^,^^32^^,^^33^ which raised fundamental questions as to whether the observed functional restoration reflected genuine cell integration. Thereafter, cell transplantation studies shifted toward cone precursor transplantation. Waldron et al. demonstrated that donor- or stem cell-derived cone precursors did integrate with the remaining ONL of cone dystrophy model mice, with the integration efficiency varying by host environment and increasing substantially in OLM-disrupted models.^37^ Subsequently, human cone precursors (week 17 of differentiation) also showed functional integration in the end-stage murine retinal degeneration models with few remaining photoreceptors (*rd1* and *Aipl1*^−/−^),^34^^,^^36^ and relatively mature cones (dd200) integrated in a cone degeneration model with remaining rods^35^ (*Cpfl1* mutant). These studies have reproducibly shown that donor cones form potential synaptic contact with host bipolar cells (as shown by immunohistology for pre- and post-synaptic markers), can develop OSs, consistently evoke light responses in host RGCs recorded by multielectrode array (MEA), and improve performance in behavior tests. The structured integration into the host ONL observed in the *Cpfl1* mutant model may suggest that the supportive framework provided by surviving host rods and Müller cells facilitates the structural stabilization and maturation of donor cones compared with the randomly oriented integration in the host retinas lacking the ONL. These findings may have promising implications for cell suspension approaches targeting cone dystrophy in human IRDs, including (1) macular degeneration with almost entire cone loss, and (2) ongoing cone degeneration with remaining rods, as illustrated in Figure 2B. These results support that cones or cone precursors can functionally integrate, both in advanced or ongoing cone degenerations, and that the host genetic background and retinal environment may be a key determinant of successful outcome.

### Challenges in cone precursor transplantation

Despite these promising results, cone precursor transplantation faces several inherent challenges. First, dissociated retinal progenitors exhibit greater immunogenicity than intact RO grafts,^60^ and metabolic stress following transplantation is an additional concern,^61^ representing potential immunological and biological vulnerabilities that require careful management in clinical settings. Second, unlike intact RO grafts, dissociated cone precursors lack an intrinsic glial scaffold of Müller glia and horizontal cells, which may compromise the structural support necessary for long-term photoreceptor survival and functional integration. Third, correct apical-basal polarity of integrated cells is not guaranteed without the support of a surviving host photoreceptor layer, although the structured integration observed in the *Cpfl1* model suggests that a remaining host ONL framework may partially compensate for this.^35^ Fourth, in the context of RP where cones undergo secondary degeneration following rod loss, whether grafted cone precursors can survive and maintain functional integration in the absence of surrounding rods remains an open question, as the trophic and structural support normally provided by the rod-dominant ONL may no longer be available at advanced disease stages. Fifth, long-term survival remains to be established: cone precursors observed by adaptive optics (AO) imaging in NHP eyes survived at the retinotomy site for up to 42 weeks,^62^ but longer-term engraftment requires further validation. With the intent of achieving stable engraftment with correct polarization of densely packed grafted photoreceptors, the transplantation using optimized scaffolds has been developed.^63^^,^^64^ Another important challenge is the clinically compatible purification of cone precursors. The above cone transplantation experiments in animals used fluorescence reporters. Identification of efficient markers for the enrichment of cones or further refinement in label-free approach are under development.^65^^,^^66^ Recently, a simple label-free procedure for both cones and rods was reported to achieve more than 90% photoreceptor enrichment by enabling the collection of the first fraction of dissociated cells from a 20-min enzymatic digestion of dd160 ROs,^67^ offering a promising option toward clinical-grade preparation.

### Preclinical evidence supporting RO transplantation for IRDs

The proof of concept for RO transplantation is rooted in earlier work on fetal retinal transplantation. Clinical studies employing fetal retinal tissue were conducted from the late 1990s through the early 2000s and yielded potentially beneficial outcomes in some patients^27^^,^^28^^,^^29^; however, ethical and practical constraints associated with fetal tissue limited the ability to draw definitive conclusions. In fetal retinal transplantation using animal models, sometimes the graft integrated with redundant retinal inner layers from both host and graft, and some synaptic connections between unspecified inner retinal cells of host and graft were suggested.^68^

Our team conducted extensive studies using mouse ROs in the end-stage *rd1* mice to explore potential and mechanism of functional photoreceptor integration in the host network. A key advantage of using RO grafts is their structural stability following transplantation and their capacity for continued maturation within a cohesive, organized cellular community in the presence of Müller glia and horizontal cells, with morphological maturation of photoreceptors with the inner segment or OSs, although photoreceptors often form rosette structures (Figures 1D and 1E). On the other hand, the general major concern was the presence of undesired retinal inner cells that may inhibit proper host-graft synaptogenesis. The principal strategy for minimizing undesired inner retinal cells in the graft is the selection of an optimal RO developmental stage. After engraftment, younger mouse ROs (i.e., dd11–17) more frequently develop laminar structures including the INL, ONL, and IPL (resulting in a laminar interception pattern) or the ONL with partial INL (direct contact pattern), whereas later-stage ROs (i.e., dd18–24) often result in loss of laminar organization (Figures 2C and 2D). This trend was consistently observed across species and differentiation stages: younger human and monkey ROs (Figures 2E and 2F, respectively), even when photoreceptors adopted rosette configurations, often developed a thicker ONL with a thicker INL and even an IPL following transplantation, while more mature ROs at the time of transplantation resulted in thinner ONL rosettes with a less-organized INL structure.^16^^,^^17^^,^^59^ This may be related to the susceptibility of early-born inner retinal cells to loss at later transplantation stages. This trade-off between structured retinal development and thinning of inner retina prompted us to use ROs at approximately dd13 (mouse) or dd55–80 (human), a stage when *CRX*^*+*^ photoreceptor progenitors emerge (Figure 1B). These grafts subsequently develop photoreceptor layers mostly adopting rosette configurations with more or less OS-like structures oriented inward, and nearly half of photoreceptor rosettes (23 of 52) establish direct contact with the host INL (Figure 2D).^16^ Notably, we consistently observed that even within rosette structures, glutamine synthase (GS)-positive Müller cells are present throughout the graft ONL, potentially supporting photoreceptor alignment *in situ*, whereas GFAP upregulation is absent specifically at the host-graft integrating portions (Figure 2G).^56^^,^^60^

To obtain unambiguous confirmation for host-graft synaptogenesis, even in the presence of graft bipolar cells, we prepared the end-stage retinal degeneration mice lacking ONL with a rod bipolar reporter (L7-GFP × *rd1*) and transplanted ROs carrying a rod synaptic ribbon reporter, pNrl:CtBP2-tdTomato, followed by immunostaining for either of the postsynaptic markers, Cacna1s or mGluR6, enabling a quantitative assessment of specific host-graft synapses (Figure 3A).^38^^,^^45^^,^^69^ The ultrastructure of these host-graft synaptic sites was further examined by correlative light and electron microscopy, revealing that, although not fully typical in morphology, these contacts assembled horizontal cells and L7-GFP^*+*^ bipolar cells in proximity to tdTomato^*+*^ synaptic ribbons suggestive of functionality.^69^ Consistently, host RGCs showed recovery of light responses in the grafted area, in accordance with the extent of the grafted area^45^ and with the presence of host-graft synapses.^69^ Transplanted mice also showed light-responsive behaviors assessed using a shuttle avoidance system.^38^^,^^45^ Another group also reported maturation and functional recovery in light responses by superior colliculus recordings and visual improvement assessed by optokinetic response (OKR) after RO transplantation in rat retinal degeneration models.^70^

**Figure 3:**
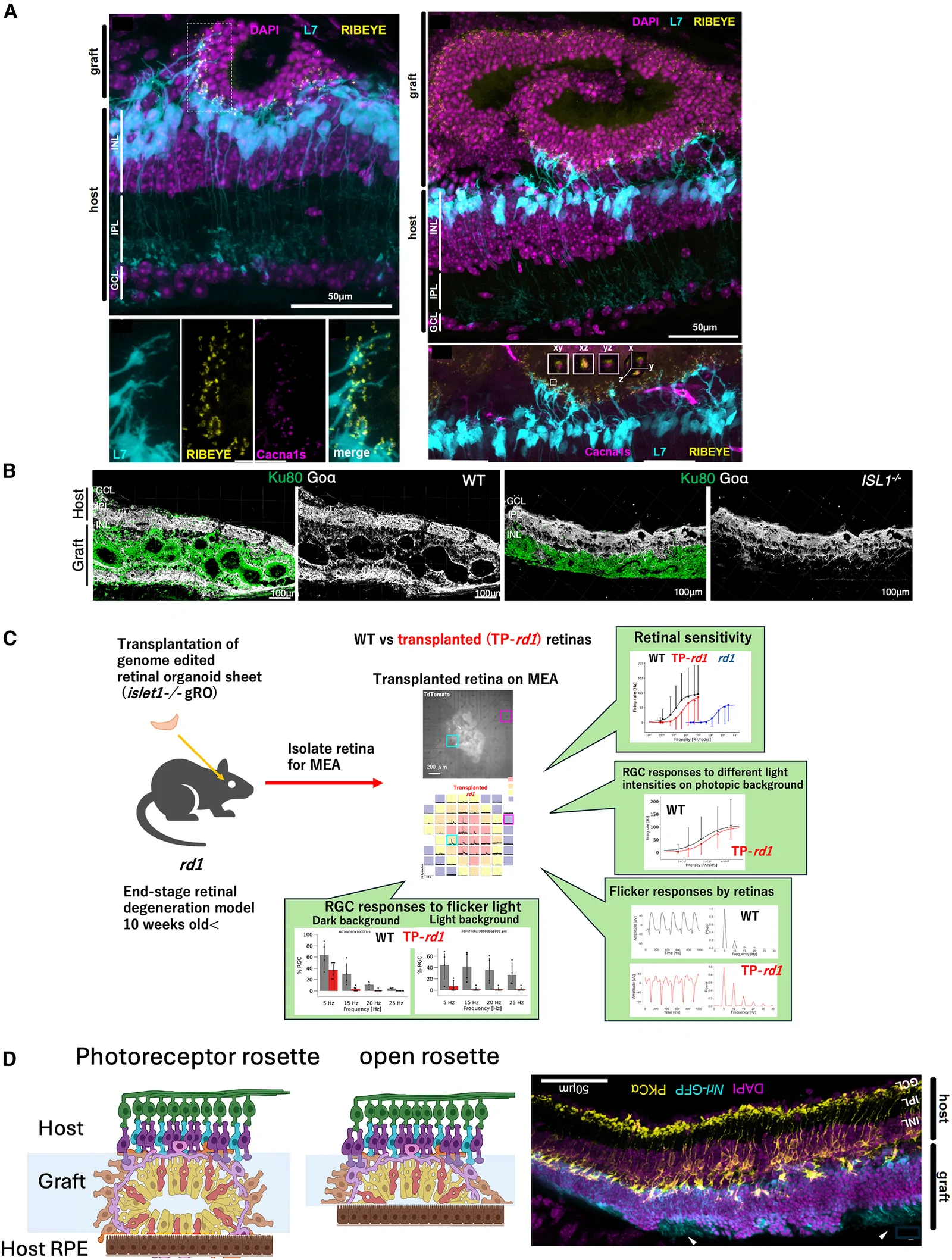
Recent advances in retinal organoid transplantation (A) Host-graft synaptic contacts were visualized using a bipolar cell reporter host mouse (*L7-GFP*/*rd1*) and synapse reporter ROs (*CtBP2-tdTomato*), with postsynaptic marker immunostaining (Cacna1s). Host bipolar cell dendrites actively extend toward grafted photoreceptors. Adapted from Matsuyama et al.^45^ (B) Immunofluorescence for Ku80 (human cell marker, green) and Goα (“on”-bipolar cells, white) in wild-type (left) and *ISL1*^−/−^(right) RO grafts. Wild-type grafts contain Goα-positive “on”-bipolar cells, whereas *ISL1*^−/−^ grafts lack these cells, enabling unambiguous visualization of host bipolar cell dendrite regrowth into the graft. Adapted from Yamasaki et al.^71^ (C) Summary of the physiological retinal function recovered in end-stage retinal degeneration retinas engrafted with *Islet1*^−/−^ mouse ROs. Transplanted retinas acquired responses to dim light within the range of normal retinal sensitivity, light-adapted responses under photopic background illumination, and responses to flicker stimuli demonstrating temporal resolution of approximately 5 Hz. Adapted from Watanabe et al.^72^ (D) Schematic comparing a closed-photoreceptor rosette and an open-rosette configuration (left), with a representative immunohistochemical image of RO rosettes in an RO graft in *rd1* mouse (right). In the open-rosette configuration, Nrl-GFP-positive graft photoreceptors show hemispherical integration with outer segments correctly oriented toward the host RPE, and horizontal cells have been observed to contribute to host-graft synapses^69^^,^^73^ (reflected in left schematic). PKCα-positive host bipolar cells form synaptic contacts with Nrl-GFP-positive grafted photoreceptors (right image). Adapted from Matsuyama et al.,^45^ the image has been vertically flipped to align orientation with the schematic illustration.

### Genome-edited RO approach for IRDs

Building on this proof of concept and with the goal of enhancing host-graft synaptogenesis, we generated ON-bipolar cell-deficient ROs by ablating genes required for bipolar cell development; first with *Islet1* and *Bhlhb4* in mice embryonic stem cells (ES cells) or iPSCs,^45^ and then in *ISL1* in human ES cells.^71^ With *Islet1*^−/−^ RO grafts in *rd1* mice, while the proportion of host rod bipolar cells forming synapses with graft photoreceptors remained comparable at approximately 14%, the number of rod synaptic contacts per host rod bipolar cell was nearly doubled.^45^ Furthermore, the absence of graft bipolar cells allowed clear visualization of host bipolar dendrite regrowth, even without the L7-GFP reporter, enabling confirmation by immunostaining alone across all host animals (Figure 3B). These dendrites reached graft photoreceptor axons and ribbons aligned on the external margin of photoreceptor rosettes in human ROs, with co-expression of multiple synaptic markers.^71^ Consistently, retinal sensitivity increased with *ISL1*^−/−^ graft compared with wild-type graft with an overall higher proportion of retinas responding to light.^71^ Potential post-transplant retinal physiological properties were further assessed in terms of host-graft network coordination by comparing wild-type retinas and severely degenerated *rd1* retinas engrafted with mouse *Islet1*^−/−^ ROs (∼19-weeks old) (Figure 3C). Post-transplant retinas demonstrated a significant increase in retinal sensitivity to almost normal retinal range, i.e., potential acquisition of dim-light vision in night blindness disease, as well as a recovery of cone function under day-light background light by adaptation, and the acquisition of rod-dominant temporal resolution in host RGC firing of approximately up to 5 Hz.^72^ These findings demonstrated that *Islet1*^−/−^ RO grafts can integrate in host retinal network in a rod-dominant manner into the residual retinal network of severely degenerated murine retinas.

Recent work also provided insights into the mechanisms underlying host-graft synaptogenesis. During normal retinal development, horizontal cells invaginate toward synaptic ribbons at photoreceptor axon terminals prior to bipolar cell ingrowth, and loss of horizontal cells leads to failure of photoreceptor synaptogenesis.^74^^,^^75^ In the absence of host horizontal cells, partly mimicking the condition of extreme end-stage retinal degeneration, graft horizontal cells were observed to recapitulate their developmental role by invaginating toward photoreceptor ribbons as synaptic “pre-sets,” and host bipolar cells subsequently completed triad synapses with grafted photoreceptors near the host-graft border.^73^ *Islet1*^−/−^ RO could functionally integrate even under these conditions, although the recovery of host RGC light responsiveness was weaker than host retinas retaining horizontal cells. This indicates that *Islet1*^−/−^ RO grafts can contribute to functional recovery, even in severely advanced retinas, though the outcome is expected to be more favorable when the host inner retina is better preserved.

### Host retinal plasticity and environment for graft integration

Importantly, although both single-cell and RO approaches have demonstrated graft potential to elicit light responses in rodents, whether the highly developed primate visual system can offer active synaptic remodeling remains a key question. The stable engraftment and the lack of ON-bipolar cells of *ISL1*^−/−^ ROs distinctly visualized active dendrite bipolar regrowth in non-human primate (NHP) eyes, spanning multiple cone and rod bipolar cell subtypes, with synaptic contact between host bipolar dendrites and graft photoreceptors confirmed by immunohistology and serial block-face scanning electron microscopy.^57^ This structural remodeling was associated with increased host ON-bipolar cell responses, as measured by focal electroretinography, indicating a meaningful capacity for synaptic rewiring in the adult primate retina. Interestingly, the proportion of cone photoreceptors in macular RO transplants in this study was substantially higher than in our previously reported mid-peripheral RO transplants (approximately 70%–80% vs. ∼15%),^17^ suggesting a possibility that the macular microenvironment may preferentially support cone survival and maturation. The macular region is distinguished by high incident light intensity, a distinct inner retinal cytoarchitecture, and unique immunological and metabolic properties, including elevated metabolic demand and a rich choroidal blood supply,^76^ each of which may have individually or collectively contributed to this cone-enriched graft outcome. Yet, this is still hypothetical, and whether the macular niche actively promotes cone dominance or selectively compromises rod integration warrants further investigation.

### Additional clinical application of RO sheets

In a distinct therapeutic context, an RO graft was applied to the treatment of NHP model of macular hole.^77^ The primary aim in this setting is anatomical closure of the retinal defect rather than network reconstruction. Intriguingly, autologous retinal patch harvested from the patients’ own peripheral fundus used for this purpose has been associated with increased retinal sensitivity within the grafted area^78^^,^^79^ and favorable visual outcomes overall,^80^ suggesting an additive contribution from the graft tissue itself. Given the invasiveness of harvesting autologous retinal tissue and the associated visual field defect at the donor site, RO patches may offer a compelling alternative to autologous retinal transplantation for this indication.

### Remaining challenges in RO transplantation

A remaining challenge in RO transplantation is the resolution of rosette structures. Although graft photoreceptors in rosette configuration often lack direct contact with the RPE, these ROs elicit host RGC light responses, even without exogenous 9-*cis* retinal in *ex vivo* MEA recordings following dark adaptation,^71^^,^^72^ indicating that graft photoreceptors retain functional capability *in vivo*. Notably, photoreceptor cells in *ISL*^−/−^ human ROs express essential phototransduction proteins as well as interphotoreceptor matrix proteins,^71^ suggesting a degree of intrinsic functional competence. This situation may bear some resemblance to central serous chorioretinopathy, in which patients generally retain relatively good vision despite disrupted photoreceptor-RPE interaction.^81^ Nevertheless, without proper apical contact between the photoreceptors and RPE, photoreceptor function is likely to remain suboptimal. We have previously observed “open rosettes” in mice and in an NHP eye, where graft photoreceptors showed hemispherical integration, with the OSs oriented correctly toward the host RPE^45^^,^^56^ (Figure 3D). Elucidating the mechanisms underlying this open rosette configuration and identifying strategies to reliably induce it will be key to improving therapeutic outcomes in RO transplantation.

## Combined deliveries for photoreceptors with RPE

In advanced retinal degeneration, RPE loss often accompanies photoreceptor degeneration, potentially limiting the maturation and function of grafted photoreceptors. One proposed strategy to address this limitation is the combined delivery of RPE cells. Lee et al. reported microfabricated, honeycomb-patterned, biodegradable poly(glycerol sebacate) (PGS) scaffolds, where they first seeded RPE cells and, 24 h later, seeded the photoreceptor cells from dd120 ROs.^82^ Another example is the preparation of the RPE sheet on the ultrathin parylene sheet and the attachment of the RO by using fibrin glue.^83^ The co-graft was successfully delivered into rat eyes with retinal degeneration, and post-transplant visual responses were recorded from the superior colliculus, likely reflecting functional integration of the RO component. At present, however, co-delivery alone does not reliably prevent rosette formation in RO grafts or ensure the structured apical orientation of graft photoreceptors toward the co-delivered RPE.

## Immunological aspects in clinical translation

The eye is intrinsically known as an immune-privileged site,^84^ with the presence of the blood-retinal barrier at retinal vessels and RPE. However, under disease conditions, these barriers are often disrupted, increasing the risk of graft rejection. Interestingly, compared with the dissociated retinal cells from ROs, ROs as whole tissue showed low immunogenicity, with a low expression of HLA molecules, immunosuppressive properties on activated lymphocyte proliferation, and the capacity to produce TGF-β2 whose immunosuppressive properties were antagonized by neutralizing antibody or the receptor inhibitor SB431542 in *in vitro* studies.^60^ Furthermore, MHC-mismatched RO transplantation in NHP showed stable graft survival and photoreceptor maturation in localized retinal degeneration with laser ONL ablation, even without systemic immunosuppression, supporting overall immune-competency of RO transplantation, although mild immune responses were occasionally observed.^59^ In a clinical study using ROs in advanced RP patients, stable graft survival was observed over 2 years with systemic immunosuppression in the first 6 months, with no apparent signs of immune rejection.^18^ This shows a sharp contrast with RPE transplantation, where MHC-mismatched RPE-strip transplantation showed significant immune rejection without immunosuppression, while MHC class II-deficient RPE strip avoided immune rejection.^85^ A clinical study using RPE-strip showed graft survival under cyclosporine during the first 6 months; however, an immune response was observed in one of three patients after discontinuation.^86^ This suggests that while a 6-month immunosuppressive regimen can effectively control early rejection, immune responses may still emerge after discontinuation, highlighting both the efficacy of the initial regimen and the need for a careful long-term follow-up with additional immunosuppressive management as needed for potentially immunologically high-risk cell therapies. Alternatively, the use of HLA-edited iPSCs^85^^,^^87^^,^^88^^,^^89^ may provide more reliable control over rejection.

In consideration of clinical practice, while the immunological requirements for photoreceptor cell transplantation are less well characterized than those for RPE transplantation, the choice of immunosuppressive strategy is likely to depend on the degree of HLA mismatch. Unlike RPE transplantation where autologous iPSC-derived grafts have been a therapeutic option, photoreceptor replacement in IRDs generally requires allogeneic sources, as patient-derived iPSCs carry the causative pathogenic variants. Gene-corrected autologous iPSCs represent a theoretical exception but remain impractical at present due to manufacturing complexity and cost; in such cases, short-term local corticosteroids to manage surgical inflammation may suffice. For allogeneic grafts with partial HLA matching, a regimen analogous to that used in the RO clinical study, including systemic immunosuppression with cyclosporine for approximately 6 months, followed by careful tapering and long-term monitoring, represents a reasonable starting framework, informed by the RPE transplantation experience. For the fully HLA-mismatched allogeneic grafts, especially with cone precursors, more intensive or prolonged immunosuppression may be required, although the inherently low immunogenicity of intact RO tissue may partially offset this risk, as suggested by the NHP data. The use of HLA-edited iPSC lines may provide a more reliable and scalable solution, reducing or potentially eliminating the need for systemic immunosuppression. Establishing optimal immunosuppressive protocols will require careful prospective evaluation in ongoing and future clinical studies.

### First-in-human clinical study using stem cell-derived RO

The first-in-human iPSC-derived RO transplantation was conducted in 2020 at Kobe City Eye Hospital.^18^ Because RO grafts are transplanted at an early developmental stage and consist of heterogeneous developing retinal progenitors, quality control (QC) focused on confirming retinal identity and developmental status, rather than the functional properties expected after maturation. Specifically, QC included assessment of continuous neuroepithelial morphology, expression of retinal lineage markers, and the absence of non-retinal markers by quantitative PCR (qPCR). QC was performed on the continuous adjacent area of each individual RO, which had been also validated as comparable in qPCR profiles to the graft sheet itself.^90^ As the transplant lacks mature photoreceptor function at the time of transplantation, these QC criteria were established based on extensive preclinical studies demonstrating that the ROs meeting these specifications consistently develop mature photoreceptors and restore light responses in host RGCs following transplantation in animal models. Establishing such surrogate QC criteria required substantial time and resources and represents one of the major translational challenges unique to progenitor-stage cell therapies at present.

In the clinical study, two advanced RP patients were recruited and 3 RO sheets each were transplanted into one eye per patient. The target site for engraftment was selected as an area retaining RPE but with photoreceptor loss, and the surgery was performed successfully. The graft survived stably for over 2 years, with AO imaging in one eye suggesting the presence of photoreceptors in rosette formations containing OS-like structures within, with an OPL-like line between the host INL and the graft, consistent with histological findings from animal studies.^91^ Although neither patient demonstrated measurable improvement on standard ophthalmological assessments such as visual acuity or Humphrey visual field testing, one patient exhibited transient improvement on three independent functional tests designed for low-vision patients (full-field stimulus testing [FST], fixation, and alphabet test). A possible hypothesis that explains the consistent improvement observed across three independent examinations at a similar time frame is that increased retinal sensitivity near the remaining central cone area may have supported fixation guidance, thereby contributing to transient improvements in alphabet recognition. This suggests that, in real-world settings, although increased retinal sensitivity alone may not appear sufficient for meaningful visual restoration, it is important to note that overall visual function reflects complex serial and collaborative processing across multiple levels of the visual system. Efficacy remains to be established in future clinical studies.

## Ongoing clinical studies aiming for photoreceptor engraftment

Preclinical evidence reviewed in the preceding sections supports two complementary approaches to photoreceptor replacement, each with distinct biological rationales and target disease profiles, as summarized in Table S1. These approaches are now being evaluated in clinical settings through two ongoing trials. One is a phase 1/2a study of subretinal administration of OpCT-001 photoreceptor precursor cells for primary photoreceptor disease by BlueRock Therapeutics (NCT06789445). The other is a phase 1/2 study of iPSC-derived RO sheet for the treatment of RP by RACTHERA and Sumitomo Pharma Co. Ltd (NCT06891885). Although the target disease population overlaps substantially, photoreceptor precursor cell suspension offers an enriched photoreceptor population with fewer non-photoreceptor cell types, while RO sheet transplantation, sharing key features with the first-in-human study conducted in Kobe, provides an intrinsic cellular scaffold of supporting cells that may facilitate photoreceptor maturation and functional integration. Given the limited understanding of immunological responses following photoreceptor precursor cell transplantation, stable long-term cell survival would be a major concern, and accordingly, safety and tolerability represent the primary endpoints, with visual function assessment as a secondary outcome. Notably, a parallel prospective observational study (NCT06517940) has been initiated by Sumitomo Pharma at Massachusetts Eye and Ear to explore optimal functional endpoints for RP, the findings of which will inform the design of future efficacy trials.

An important challenge shared by both approaches is the lack of established methods to evaluate graft maturation status *in vivo*, which is a prerequisite for any therapeutic effect, as both strategies involve transplantation of progenitor-/precursor-stage grafts or cells. Conventional electrophysiology as a subsequent functional readout may not be sufficiently sensitive to detect light responses when the number of photoreceptor cells is limited and spatially localized. At present, most informative *in vivo* assessment would be AO imaging including optoretinogram,^92^ which has the potential to visualize functional photoreceptors—but only if correctly oriented with the developed OSs—as evidence of functional maturation.

A further challenge is that the expected functional outcomes and their magnitude remain unclear. In fetal retinal transplantation, as a sheet^27^ or as microaggregate suspensions, potential improvements in retinal sensitivity or subjective vision were reported in some patients,^28^^,^^29^ suggesting that even modest photoreceptor integration may yield measurable functional benefits. The appropriate endpoints for future efficacy trials are discussed in the following section.

## Translating preclinical efficacy into clinical benefit

The restoration of complex visual function in the highly developed primate visual system is inherently challenging. While incremental advances in graft survival, photoreceptor maturation, and basal functional integration have been demonstrated, how well these are integrated into the degenerating host retinal network remains unclear, given that the retina constitutes a complex network that coordinates the final visual output. Stepwise therapeutic improvement, therefore, may represent the most realistic clinical development path, and the most relevant efficacy markers are likely to evolve accordingly, shifting from basic measures of retinal sensitivity toward higher-order visual function assessments that better reflect meaningful patient benefit.

A critical challenge for the clinical development of photoreceptor transplantation is establishing endpoints that meaningfully capture treatment benefit in patients. In animal models, efficacy is typically evaluated using objective measures of retinal or visual pathway function, including MEA recordings, optomotor responses, and light-avoidance behavior. These assays provide evidence that transplanted photoreceptors survive, integrate into host retinal circuits, and contribute to visual signal transmission. The corresponding endpoints in human studies include best-corrected visual acuity (BCVA), microperimetry, visual field testing, and FST. Similar to preclinical assays, these measures primarily quantify the retinal function and visual sensitivity. However, unlike animal studies, clinical trials must address whether biological restoration translates into meaningful improvements in daily life. As recognized across regenerative medicine, surrogate physiological endpoints do not always predict outcomes that matter most to patients.

This concept has already influenced outcome assessment in IRD trials. The development of voretigene neparvovec (Luxturna), which is a gene therapy for *RPE65*-associated retinopathy, demonstrated the value of functional vision testing through the multi-luminance mobility test (MLMT),^93^ while newer tools such as the multi-luminance Y-mobility test (MLYMT) and multi-luminance shape detection test (MLSDT) have further expanded the ability to quantify vision-dependent daily activities.^94^ Patient-reported outcomes (PROs) are also increasingly being recognized as essential measures of treatment benefit.

An important consideration specific to photoreceptor cell therapy is that rod and cone reconstructions are expected to yield distinct functional benefits, which should be reflected in endpoint selection. Successful rod integration may increase retinal sensitivity and improve vision under dim light, best captured by FST, as suggested by our previous RO clinical study^18^ and RPE transplantation in a patient with *RPE65-*associated retinopathy.^95^ FST can be supplemented by microperimetry for localized sensitivity mapping at the graft site and fixation stability testing to capture higher-order collaborative visual processing. Mobility tests would further extend this to real-world functional vision. Flicker testing or motion detection tasks such as bar movement recognition would help characterize cone-rod contributions following engraftment.^72^ In contrast, although a better utilization of remaining cones near the central fovea would be expected to improve visual resolution including letter recognition and reading ability, how cone reconstruction by engrafted cones may contribute to such outcomes remains to be elucidated in future clinical studies. PROs would further complement these objective measures by capturing vision-related quality of life improvements that may not be fully reflected in standard assessments.

These considerations suggest a hierarchical framework for future photoreceptor replacement trials; optoretinogram or retinal sensitivity measures such as FST and microperimetry serve as indicators of photoreceptor function and integration; fixation stability and mobility- and navigation-based assessments serve as measures of functional visual behavior; and reading performance or other higher-order visual tasks capture patient-relevant benefit. The optimal endpoint strategy will depend on the disease stage and treatment target. Extramacular RO sheet transplantation may be particularly suited for patients with advanced rod degeneration and preserved central cone vision, whereas cone-focused cell replacement strategies may target patients with central visual impairment. Whether such stepwise progression will ultimately translate into vision restoration that is meaningful in daily life, ranging from barely supportive functional gains to substantial improvements such as reading ability, remains to be seen. Realizing this potential requires appropriate endpoint selection that integrates physiological, behavioral, and PRO measures. Equally important is a regulatory and clinical framework that accommodates the stepwise nature of this emerging field.

## Beyond biology: Manufacturing, regulatory, and clinical challenges

Beyond biological challenges, successful clinical translation also requires overcoming several manufacturing and regulatory hurdles. One of the major regulatory challenges for RO transplantation is the lack of a validated potency assay for a graft composed of heterogeneous developing cells. Because ROs are transplanted at an immature progenitor stage, direct functional testing before transplantation is currently not feasible, and the release criteria rely on retinal identity, morphology, and lineage marker expression. Yet, the reproducible and specific morphology of ROs is itself indicative of a coordinated cellular organization, wherein specific cell types at defined developmental stages collectively contribute to the overall tissue architecture, potentially securing the developmental trajectory of the graft once placed in the ocular environment. *In vivo* functional validation at every developmental step before establishing QC criteria would demand years of clinical verification per iteration, rendering efficient therapeutic advancement impractical. Identifying robust QC indicators grounded in morphological and molecular characteristics will be essential, and in our view, a regulatory framework that recognizes RO-specific morphology as a meaningful surrogate for potency, where feasible, will substantially facilitate stepwise clinical development. Emerging tools such as transcriptomic profiling and computational morphology analysis may further support the scientific rationale underlying such criteria, even if not directly incorporated into routine QC workflows.

Scalable manufacturing of clinical-grade RO grafts and photoreceptor precursor donor cells also requires the establishment of good manufacturing practice (GMP)-compliant master iPSC cell banks (MCBs). MCB qualification typically encompasses donor infectious disease screening, sterility, endotoxin, and mycoplasma testing, as well as iPSC-specific characterization including identity, purity, and genomic integrity, in accordance with the EMA and FDA regulatory frameworks.^96^ An additional practical consideration is the cryopreservation of grafts to enable off-the-shelf availability and logistical flexibility between manufacturing and transplantation sites. Unlike terminally differentiated cells such as RPE, where cryopreserved cells have been used in clinical studies,^97^^,^^98^ progenitor-stage grafts exist in a dynamic state of active differentiation that complicates cryopreservation, in which individual cells are in heterogeneous and transient developmental stages. Resumption of coordinated differentiation after thawing cannot be assumed for all cells, and recovery culture prior to transplantation is likely essential to restore graft quality and developmental trajectory. Furthermore, cryopreservation-induced cellular stress may alter the immunogenic profile of grafts, potentially affecting immune responses after transplantation; this aspect has not been systematically characterized for retinal progenitor-stage grafts and represents an additional consideration for clinical translation.^99^^,^^100^ In our experience, cryopreservation of RO grafts resulted in impaired post-transplant function; we, therefore, established a non-cryogenic preservation protocol for maintaining RO grafts at 17°C for 3–4 days, and the protocol was validated by confirming preserved light-evoked responses in host RGCs by MEA following transplantation in rats and subsequently adopted for our clinical study.^90^ Validating cryopreservation and recovery protocols that maintain graft developmental competence and ultimately support functional integration after transplantation remains an important unresolved challenge for clinical scalability.

## Overall conclusions and future perspectives

The studies reviewed here illustrate the remarkable and ongoing progress in photoreceptor replacement therapy using iPSC-derived retinal cells and organoids. From early mechanistic studies of host-graft interactions to the first clinical application of iPSC-derived RO transplantation, growing evidence supports that grafted photoreceptors can form genuine synaptic connections with host retinal circuits and contribute to functional recovery. The success of this approach is profoundly shaped by the residual host retinal architecture, disease stage, and regional niche properties, as exemplified by the cone-favoring environment of the macula. Looking forward, continued advances in genome editing, scalable biomanufacturing, and refined transplantation techniques, combined with rigorous clinical evaluation, offer a potential path toward vision restoration for patients with currently untreatable retinal degenerative diseases.

## Declaration of interests

M.M. received research funding from Sumitomo Pharma Co. Ltd. for the past clinical study. M.M. and A.M. have received research funding from VCCT Inc. M.M. also has a pending patent application related to genome-edited ROs for transplantation therapy.

## Supplemental information

Document S1. Table S1

Document S2. Article plus supplemental information
