# Supplementary material for: iPSC-derived retinal organoids: Biological insights and progress toward photoreceptor replacement therapy
Source: Cell Rep Med. 2026 Sep 25;7(10):103053. doi: 10.1016/j.xcrm.2026.103053 (PMC13710147; doi:10.1016/j.xcrm.2026.103053)
Supplement: Supplementary file 1 [file mmc1.pdf]

Cell Reports Medicine, Volume *7*

Supplemental information

iPSC-derived retinal organoids: Biological insights and progress toward photoreceptor replacement therapy

Akiko Maeda, Xiaoxu Han, and Michiko Mandai

Supplementary Table

Comparison between cone and RO transplantation in preclinical studies

	Cone transplantation	RO transplantation
Target disease	・Cone dystrophy<br>・Macular degeneration	・Primary rod degeneration (retinitis pigmentosa)<br>・End-stage macular degeneration
Expected functional outcomes	・Cone-mediated function (potentially improved resolution suggested by OKR).<br>・No evidence of color vision restoration to date	・Rod-mediated function (increased retinal sensitivity; potentially perception of movement suggested by temporal resolution assessed by MEA)<br>・Cone-mediated function (potentially improved resolution suggested by OKR and b-wave increase in NHP focal ERG)<br>・No evidence of color vision restoration to date
Graft culture stage	dd100-dd200	dd55-dd90
Graft preparation	・Precursor/cone enrichment procedure required	・Simple excision (requires expert skill)
Graft composition	・Cone precursors to relatively mature cones	・Photoreceptors, horizontal cells, Müller glia (RGC, Amacrine, ON-bipolar cells are reduced in *ISL1*$^{-/-}$ grafts)
Advantages	・Enriched population of therapeutically relevant cells	・Müller glia and horizontal cells may support photoreceptor survival and integration<br>・Low immunogenicity
Limitations	・Potential immunological vulnerability<br>・Lack of intrinsic glial scaffold<br>・Apical-basal polarity not guaranteed without host ONL support<br>・Uncertain survival without surrounding rod photoreceptors<br>・Long-term engraftment not yet validated in primates	・Mixed retinal cell population may interfere with synaptogenesis (*ISL1*$^{-/-}$ grafts reduce this risk)<br>・Rosette formation limits photoreceptor-RPE interaction<br>・Strategies to reliably induce open rosette configuration are still under development
